# A Compact and Smooth CH_3_NH_3_PbI_3_ Film: Investigation of Solvent Sorts and Concentrations of CH_3_NH_3_I towards Highly Efficient Perovskite Solar Cells

**DOI:** 10.3390/nano8110897

**Published:** 2018-11-01

**Authors:** Liang Chen, Hao Zhang, Jiyuan Zhang, Yong Zhou

**Affiliations:** 1 Hunan Key Laboratory of Applied Environmental Photocatalysis, Hunan Collaborative Innovation Center of Environmental and Energy Photocatalysis, Changsha University, 98 Hongshan Road, Changsha 410022, China; zhanghao0122@126.com; 2 Jiangsu Key Laboratory for Nano Technology, National Laboratory of Solid State Microstructures, College of Engineering and Applied Sciences, and Collaborative Innovation Center of Advanced Microstructures, Nanjing University, 22 Hankou Road, Nanjing 210093, China; zhouyong1999@nju.edu.cn; 3 Kunshan Innovation Institute, Nanjing University, Kunshan, Jiangsu 215347, China; jyzxni@163.com

**Keywords:** solvent, compact, smooth, perovskite solar cells

## Abstract

Four solvents (isopropanol (IPA), *n*-butyl alcohol (NBA), *n*-amyl alcohol (NAA), and *n*-hexyl alcohol (NHA)) were investigated to prepare CH_3_NH_3_I (methylammonium iodide, MAI) solutions to transform PbI_2_ film into CH_3_NH_3_PbI_3_ (MAPbI_3_) film. It was found that the morphology of the perovskite MAPbI_3_ film was not only affected by the chain of the solvent molecule, but also by the concentration of MAI. The use of solvents with a long alkyl chain (NAA and NHA) allowed the MAPbI_3_ to grow via an *in situ* transformation step, which easily made the perovskite films compact, but with a high surface roughness due to the growth of unexpected nanorods/nanoplates. The solvent with a short alkyl chain (IPA) led to the dissolution−crystallization growth mechanism, resulting in rapid generation of perovskite films with a number of pinholes. A high-quality (compact, smooth, pinhole-free) perovskite film was obtained with NBA and an optimized MAI concentration of 8 mg/mL. The corresponding perovskite solar cells achieved a maximum power conversion efficiency (PCE) of 16.66% and average PCE of 14.76% (for 40 cells).

## 1. Introduction

Organic-inorganic lead halide perovskite solar cells have attracted intensive attention due to the remarkable progress in power conversion efficiency (PCE), increasing rapidly to over 22% in a few years [1,2,3,4,5,6,7,8,9,10,11,12,13,14,15,16,17,18]. The PCEs of perovskite solar cells with various architectures (mesoporous or planar heterojunction structures) are strongly dependent on the morphology of the perovskite film [19,20,21,22,23,24,25,26]. The growth mechanism relating to the chemical reaction kinetics significantly influences the perovskite polymorph [27]. There are several fabrication methods of perovskite films, including two-step deposition techniques [6,7], one-step solution spin-coating [8,28,29], and vacuum-evaporation deposition [9,30]. Among them, the two-step solution-process deposition technique is an easy method to obtain cells with excellent photovoltaic performance in a reproducible way [6,7]. 

In a typical two-step sequential solution process, PbI_2_ film is first deposited on a TiO_2_-coated substrate by spin-coating with a dimethylformamide (DMF) solution of PbI_2_, and is subsequently transformed into a perovskite CH_3_NH_3_PbI_3_ (methylammonium lead iodide, MAPbI_3_) film through exposure to a CH_3_NH_3_I (methylammonium iodide, MAI) solution in isopropanol (IPA) [6,7]. However, this two-step solution method often results in poor grain structure, incomplete surface coverage, and high surface roughness [19,20,21,22,23,25], which limits the further improvement of photovoltaic performance of perovskite solar cells. In a previous investigation, it was proposed that the perovskite grain morphology was governed by its growth mechanism. The growth mechanism is in line with a competing relation between an *in situ* transformation and dissolution-crystallization mechanism, which is determined by the rate and amount of PbI_2_ dissolving in the MAI solution [27]. It was confirmed that the MAI concentration significantly affects the solubility of PbI_2_ in the MAI solution and the morphology of perovskite grains. When the MAPbI_3_ crystals grow based on the *in situ* transformation mechanism, the formed MAPbI_3_ grains will maintain parallel geometries to PbI_2_. On the other hand, in the dissolution–crystallization mechanism, the large amount of dissolved PbI_2_ results in the rapid generation rate of perovskite grains, the collapse of lead backbones, and the occurrence of tetragonal-shaped MAPbI_3_ [27]. Hence, perovskite films forming in different solutions might display diverse morphologies. Unfortunately, the role of the solvent on the MAPbI_3_ film formation has been neglected. Thus, it is essential to systematically probe the relationship between the chemical properties of the solvent and the perovskite crystal growth mechanism. 

Herein, besides commonly used IPA, *n*-butyl alcohol (NBA), *n*-amyl alcohol (NAA), and *n*-hexyl alcohol (NHA) (the corresponding molecular formula are shown in Figure S1 in Supporting Information) were also selected as solvents to prepare MAI solutions to transform PbI_2_ into MAPbI_3_. From IPA to NHA, the polarity of the solvents decreases with the increasing length of the molecule chain. Considering that PbI_2_ is a polar molecule, its solubility is oppositely correlated with the length of the alkyl chain of the solvent molecule in the MAI solution. Therefore, the effect of the length of the alkyl chain of the solvents on the perovskite film morphology could be investigated systemically. 

In this work, it was found that the morphology of the perovskite MAPbI_3_ film was significantly affected by the alkyl chain of the solvent (i.e., solvent polarity) and the MAI concentration. On one hand, the coverage and compactness of the perovskite film was improved through the use of a solvent with a longer alkyl chain of MAI. This may be attributed to the slow chemical reaction rate of PbI_2_ and MAI, resulting from the low polarity of the solvent with a longer alkyl chain. On the other hand, the long-alkyl-chain solvent may also generate unexpected nanorods/nanoplates on the film surface that increase the roughness of the perovskite film, which is negatively related to the photovoltaic performance of the perovskite solar cell. In a word, both the high concentration of MAI and long reaction time facilitated PbI_2_ to form PbI_3_^-^ or PbI_4_^2-^, which also led to large crystal MAPbI_3_ nanorods/nanoplates growing on the surface with long loading time [27,31]. The best-quality (highly compact, pinhole-free, and smooth) perovskite films were fabricated with an optimized MAI concentration of 8 mg/mL and using NBA as solvent. The resulting perovskite solar cell based on this film achieved a maximum PCE of 16.66% and average PCE of 14.76% (for 40 cells).

## 2. Experimental Section

### 2.1. Materials

Methylamine (33 wt% in absolute ethanol), hydroiodic acid (57 wt% in water, 99.99%), and Li-bis(trifluoromethanesulfonyl) imide (Li-TFSI) were purchased from Sigma-Aldrich (St. Louis, MO, USA). 4-tert-butylpyridine (TBP) was purchased from Aladdin (Shanghai, China). Acetonitrile (99.8%), chlorobenzene (99.9%), dimethylformamide (DMF) (99.9%), PbI_2_ (99.999%), IPA, NBA, NAA, and NHA were purchased from Alfa Aesar (Ward Hill, MA, USA). 2,2’,7,7’-tetrakis-(*N*,*N*-di-p-methoxyphenylamine)-9,9’-spirobifluorene (spiro-OMeTAD) (≥99.0%) was purchased from Shenzhen Feiming Science and Technology Co., Ltd. (Shenzhen, China). 

### 2.2. MAI Preparation

A solution was prepared by reacting methylamine (27.8 mL, 33 wt% in absolute ethanol) with hydroiodic acid (30 mL, 57 wt% in water) at 0 °C for 2 h. Subsequently, a precipitate was obtained through rotary evaporation of the solution at 60–70 °C. The as-prepared product was washed with diethyl ether and ethanol, and then dried in a vacuum oven at 60 °C overnight.

### 2.3. Devices Fabrication

A TiO_2_ compact layer was prepared on fluorine-doped tin oxide glass (FTO) by spin-coating a sol-gel solution at 5000 rpm for 30 s and sintering at 500 °C for 30 min. The sol-gel solution consisted of 18 ml ethanol, 1.8 mL tetrabutyl titanate, and 0.38 mL diethanolamine. Subsequently, the TiO_2_ microstructure film was fabricated by spin-coating with dispersion (Deysol HR TiO_2_/ethanol 1:5 wt) and heated at 500 °C for 30 min.

Next, 60 μL of 1.0 M PbI_2_ solution in DMF was dropped on the TiO_2_-coated FTO substrate, and the substrate was then spun at 4000 rpm for 25 s. The as-prepared PbI_2_ film was heated at 70 °C for 10 min, and then naturally cooled to room temperature. Subsequently, 150 μL MAI solution was used to fully covered the PbI_2_ film. After a certain loading time (1–3 min), PbI_2_ was transformed into MAPbI_3_, and the solution was then dried by spinning. The MAPbI_3_ film was heated at 100 °C for 30 min. When the MAPbI_3_ film cooled down to room temperature, 30 μL of hole-transport materials (HTM) solution was spun on the film at 3000–4000 rpm for 45 s. The HTM solution was prepared by dissolving 68 mM spiro-OMeTAD, 26 mM Li-TFSI, and 55 mM TBP in acetonitrile and chlorobenzene (*v*/*v* = 1:10). The above processes were operated in an Ar glove box. Finally, a back-contact electrode (120 nm silver film) was evaporated on HTM layer. In this work, the active area of a cell was 0.09 cm^2^. A series of solar cells were prepared by tuning the solvents and concentration of MAI in different MAI solution. The solvent sorts are IPA, NBA, NAA and NHA; the concentrations of MAI in the four-selected solvents range from 6 to 12 mg/mL.

### 2.4. Characterization

The crystallographic phases of the as-prepared products were determined by powder X-ray diffraction (XRD) (Rigaku Ultima III, Tokyo, Japan) using Cu-Ka radiation (*λ* = 0.154178 nm) with a scan rate of 10 °/min at 40 kV and 40 mA. The morphologies of the films were examined with a field emission scanning electron microscope (FE-SEM) (JEOL, Tokyo, Japan, JSM-6700F with an accelerating voltage of 5 kV). Atomic force microscope (AFM) analysis was performed on a MFP3D microscope (Asylum Research, MFP-3D-SA, Santa Barbara, CA, USA). The absorption properties of films were investigated by ultraviolet-visible (UV-vis) spectrometer (Shimadzu UV-2550, Kyoto, Japan) and all the data were corrected by deducting the baseline value of the TiO_2_-coated FTO substrate. Photocurrent density-voltage (I-V) measurements were carried out with a Keithley (Johnston, OH, USA) 236 source measurement unit under AM 1.5 illumination cast with an Oriel 92251A-1000 sunlight simulator (Irvine, CA, USA) calibrated with the standard reference of a Newport 91150 silicon solar cell. The external quantum efficiency (EQE) spectra were measured under monochromatic irradiation with a xenon lamp and a monochromator. Photoluminescence (PL) spectrum was obtained with a 532 nm pulsed laser as excition source at a frequency of 9.743 MHz.

## 3. Results and Discussion

**Figure 1:**
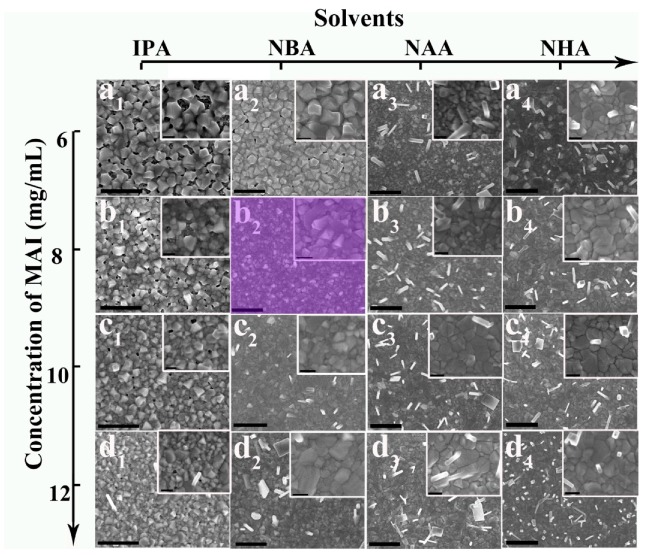
Field emission scanning electron microscope (FE-SEM) images of CH_3_NH_3_PbI_3_ (MAPbI_3_) films obtained from 6–12 mg/mL CH_3_NH_3_I (MAI) in (**a_1_**–**d_1_**) isopropanol (IPA), (**a_2_**–**d_2_**) *n*-butyl alcohol (NBA), (**a_3_**–**d_3_**) *n*-amyl alcohol (NAA), and (**a_4_**–**d_4_**) *n*-hexyl alcohol (NHA). The scale bars of **a_1_**–**d_4_** are 1 μm, and those of inserts are 200 nm.

Figure 1a_1_–a_4_ shows the morphologies of various perovskite films prepared using different solvents with the same MAI concentration of 6 mg/mL. As the solvent changes from IPA to NHA accompanying with the alkyl chain increasing, the perovskite films become pinhole-free and compact. The presence of the pinholes may result in subsequently coated HTM solutions to deleteriously infiltrate into the perovskite absorber layer. It increased the recombination of electron and hole due to the large contact area between HTM and perovskite [13]. Furthermore, for NAA and NHA solvents, some nanorods/nanoplates were observed to grow on the film surface, which will be detrimental to the performance of the perovskite solar cell. The presence of the nanorods/nanoplates requires thick HTM layers to cover the perovskite in order to eliminate the short-circuiting [22]. Thus, the thick HTM layer may result in lower short-circuit photocurrent density (*J*_sc_) and fill factor (FF) due to the low conductivity of the HTM. 

Through careful observation of the film (inserts of Figure 1a_1_–a_4_), the perovskite grains were found to gradually transit from nanocuboids to an unspecific shape without any corners. The morphology change trend indicated different growth mechanisms of perovskite grains between the four used solvents. For the *in situ* transformation mechanism, the resulting perovskite grains should keep parallel geometries to PbI_2_ due to the preservation of the inorganic lead framework. For the dissolution-crystallization mechanism, grains would be reconstructed into a tetragonal shape because of the tetragonal phase MAPbI_3_ [27]. In order to clearly reveal the effect of the solvent molecule, we specifically explored the growth mechanisms of the perovskite grains in IPA and NHA solutions. Two series of films were fabricated through exposing PbI_2_ films to the 6 mg/mL MAI in IPA or NHA with a loading time of 0–3 min. Note that the 0 min loading time signifies that the film was quickly dried through spinning as soon as the PbI_2_ film was covered with the MAI solution. X-ray diffraction (XRD) patterns (Figure S2) and ultraviolet-visible (UV-vis) absorption spectra (Figure S3) were performed to trace the reaction process of the PbI_2_ film with MAI in IPA and NHA. In the case of IPA, only the film prepared with a 0-min loading time shows a PbI_2_ diffraction peak, whereas no PbI_2_ peak was observed in the other films with longer loading times (Figure S2a). This demonstrates that the reaction of PbI_2_ and MAI can be finished in 1 min in IPA. The smaller differences between the UV-vis absorption spectra of those films with loading times of 1–3 min for IPA also demonstrate that the PbI_2_ film was totally transformed into the MAPbI_3_ film in 1 min (Figure S3a). The dissolution-crystallization mechanism is responsible for the fast reaction [18]. The PbI_2_ peak intensity of the film in NHA was gradually decreased with increasing loading time, and totally disappeared at 3 min (Figure S2b). This means that the formation of the PbI_2_ residue-free perovskite film took 3 min of loading time. The UV-vis absorption spectra of those films showed that the absorption obviously increased at 2 min, and differences decreased between 2 and 3 min in NHA (Figure S3b), which indicated that the formation of MAPbI_3_ film was completed in 2–3 min. The slower rate of perovskite film generation provides evidence for the *in situ* transformation mechanism in NHA [27]. In addition, long reaction times (2–3 min) would make a certain amount of PbI_2_ dissolve in NHA, resulting in the formation of perovskite nanorods/nanoplates.

The corresponding FE-SEM images of the resulting films are presented in Figure S4. For IPA, the number of perovskite nanocuboids increased with loading time from 0 min (Figure S4a) to 1 min (Figure S4b), and the interconnection between nanocuboids became closer. The morphologies of films with 2 min (Figure S4c) and 3 min loading (Figure S4d) are not obviously different from that of 1 min loading, and the films still have incomplete coverage. The final tetragon-shaped morphology (Figure S4a–d) implies the collapse of the lead backbone occurred in the IPA solution with the present 6 mg/mL MAI. This indicates that the growing MAPbI_3_ crystals followed a dissolution-crystallization step. Notably, the randomly stacked nanocuboids might be responsible for difficulties in obtaining pinhole-free and full-coverage perovskite films from IPA solutions.

For NHA, with 0 min loading time, the film was found to be already full-covered but the grains did not interconnect closely (Figure S4e). The interconnection became increasingly closer with the extension of loading time, and the perovskite films finally became very compact (Figure S4f–h). The applied PbI_2_ films consisted of unspecific-shaped particles (Figure S5), and the subsequent perovskite grains of the full-covered films were still unspecific-shaped rather than tetragonal. This indicated the dominant growth mechanism was *in situ* transformation in the NHA solution with the present 6 mg/mL MAI [27]. With the *in situ* transformation, the MAPbI_3_ grains grow up *in situ* to fill the void between the pristine PbI_2_ particles (Figure S5), resulting in the film easily becoming compact and complete. However, perovskite nanorods grow upon the surface with loading times of 2 min or longer. The reason is that PbI_2_ is partly soluble in the MAI solution by forming a Pb complex (PbI_3_^−^ or PbI_4_^2−^), and the dissolving Pb complex increases with longer loading times so that large and tetragon-shaped MAPbI_3_ crystals grow on the surfaces of underlying film via the recrystallization step [27,31], i.e.,

$${\mathrm{PbI}}_{2}+xI^{-}⇌{\mathrm{PbI}}_{2+x}^{x-}(x=1,2)$$

$${\mathrm{CH}}_{3}{\mathrm{NH}}_{3}^{-}+{\mathrm{PbI}}_{2+x}^{x-}⇌{\mathrm{CH}}_{3}{\mathrm{NH}}_{3}{\mathrm{PbI}}_{3}+(x-1)I^{-}(x=1,2)$$

Therefore, the *in situ* transformation mechanism occurred in NHA solution, whereas the dissolution-crystallization mechanism appeared in the IPA solution. It could be concluded that the molecules with a longer alkyl chain gave rise to slower chemical reaction rates. The results might be ascribed to the polarity of NHA being lower than that of IPA due to the different alkyl-chain lengths. Given that PbI_2_ is a polar molecule, it easily dissolves in the IPA solution, and then forms nanocuboids of MAPbI_3_. For NHA, the *in situ* transformation mechanism might bear responsibility for the formation of full-covered film, as the grain gradually grows *in situ* and maintains the inorganic lead framework (unspecific-shaped PbI_2_ particles). In other words, the coverage and compactness of the perovskite film could be easily achieved by using a longer-alkyl-chain solvent (i.e., low polar solvent) for MAI. 

The morphology of the perovskite MAPbI_3_ film does not only depend on solvent polarity but also on the MAI concentration. Therefore, the relationship between the concentration of MAI and quality (e.g., compactness and smoothness) of the MAPbI_3_ film was also studied. The surface morphologies of the various MAPbI_3_ films obtained from different concentrations of MAI are described in Figure 1 and summarized in Table 1. The obtained perovskite films could be classified into three basic types: (1) incomplete-covered film, (2) compact film roughed with nanorods/nanoplates, and (3) compact and smooth film. All the perovskite films were proved to be PbI_2_-free by XRD patterns (Figure S6). For IPA, the resulting MAPbI_3_ films were incomplete-covered (Figure 1a_1_–d_1_). With increasing MAI concentrations, the coverage of the perovskite film increased, but the size of the perovskite grains decreased. These findings are in agreement with the literature [1,27]. No perovskite nanorods grew on the surface of films except sporadically on the film obtained from the 12 mg/mL MAI in IPA. For NBA, the perovskite grains growing in 6 mg/mL MAI were observed to still be nanocuboids (Figure 1a_2_). Although the perovskite film contained a number of pinholes, its coverage was better than that with the IPA solution. The film prepared with 8 mg/mL MAI in NBA became very compact, and no obvious perovskite nanorods were observed (Figure 1b_2_). Thus, this pinhole-free film was quite smooth. Since the high-quality film consisted of perovskite grains with tetragonal and unspecific morphologies, both dissolution–crystallization and *in situ* transformation mechanisms worked together with 8 mg/mL MAI in NBA. Perovskite nanorods or nanoplates began to form on the surfaces of two films obtained from 10 and 12 mg/mL MAI in NBA (Figure 1c_2_,d_2_). Figure 1a_3_–d_4_ shows that the surfaces of all compact films prepared with 6–12 mg/mL in NAA or NHA also contained nanorods or nanoplates. These nanorods/nanoplates grew on the compact film surface through CH_3_NH_3_^−^ reacting with the Pb complex (PbI_3_^–^ or PbI_4_^–^) in solutions of MAI, increasing the surface roughness. Figure S7 shows the corresponding atomic force microscope (AFM) images and root mean squares (RMS) of three perovskite films respectively obtained from 6, 8, and 10 mg/mL MAI in NBA, which represented incomplete-covered film, smooth and compact film, and compact film roughed with nanorods/nanoplates, respectively. With the film changing from incomplete to compact, the RMS decreased due to the disappearance of pinholes. Figure S7c indicates that the increase of the RMS originates from the height of nanorods/nanoplates over the compact film surface.

**Figure 2:**
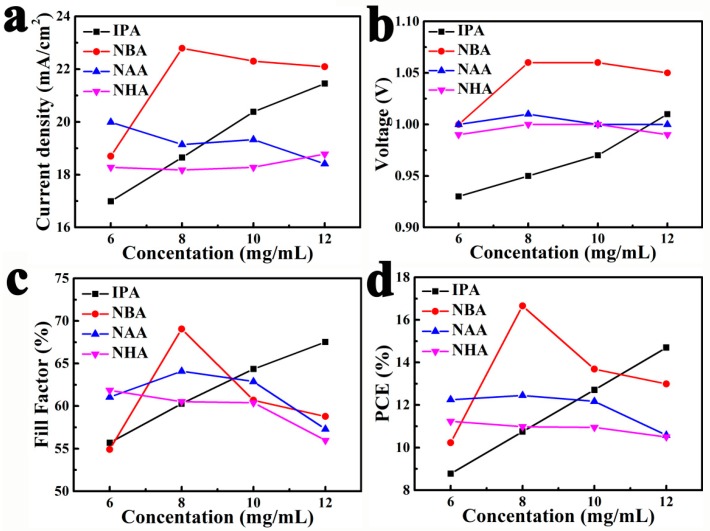
Detail parameters and power conversion efficiencies (PCEs) of the cells prepared by using MAPbI_3_ films obtained from 6–12 mg/mL MAI in IPA, NBA, NAA, or NHA.

**Figure 3:**
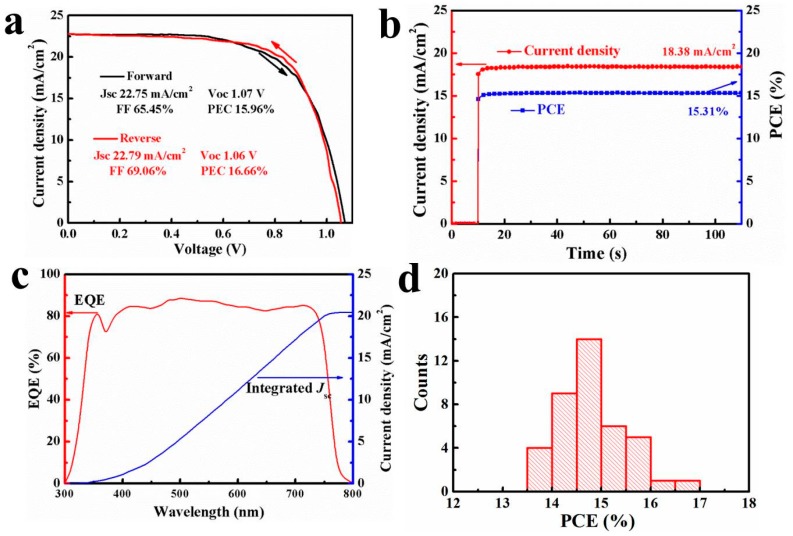
(**a**) Density-voltage (I–V) cures of the best-performing cell, the voltage scan rate is 50 mV/s (**b**) Photocurrent density and PCE as a function of time for the same cell under 0.833 V, (**c**) External quantum efficiency (EQE) spectrum of the best-performing cell, (**d**) Histograms of PCEs measured for 40 cells prepared by using 8 mg/mL MAI in NBA.

A series of perovskite solar cells were assembled to study the photovoltaic performance of those PbI_2_ residue-free perovskite films. Figure 2 performs the photovoltaic parameters of the corresponding devices as functions of the MAI concentrations and solvent sorts. Those photovoltaic parameters were obtained from photocurrent density-voltage (I-V) measurements under reverse scan. On one hand, the perovskite solar cells obtained from 6–8 mg/mL MAI in IPA suffered lower *J*_sc_ (16–18 mA/cm^2^) and open-circuit voltage (*V*_oc_, 0.93–0.95 V) due to serious recombination. The reason for this is that HTM infiltrated into the incomplete-covered films through the pinholes (Figure 1a_1_,b_1_), which increased the contact area between HTM and perovskite. The higher *J*_sc_ (20–21 mA/cm^2^) and *V*_oc_ (0.97–1.01 V) of the films from 10–12 mg/mL MAI in IPA resulted from the improvement of perovskite film coverage (Figure 1c_1_,d_1_). On the other hand, unsatisfactory and slightly different PCEs (range 10–12%) were achieved by those cells obtained from NAA and NHA solutions, resulting from lower *J*_sc_ (17–19 mA/cm^2^) and fill factor (FF, 54–64%). This might be explained by the fact that thick HTM layers were required to completely cover the compact perovskite films with dense nanorods/nanoplates (Figure 1c_2_–d_4_ and Figure S7c), which increased the series resistance of devices. A solar cell obtained from an NBA solution of 8 mg/mL MAI (Figure 1b_2_) achieved promising *J*_sc_ (22.79 mA/cm^2^), *V*_oc_ (1.06 V), FF (69%), and PCE (16.66%), which is ascribed to the compact and smooth perovskite film without pinholes or nanorods/nanoplates. In addition, it is well known that perovskite solar cells often show an unusual hysteresis in the I-V curves. All perovskite solar cells were measured with both reverse and forward scan directions to estimate their photovoltaic performance. The corresponding detailed parameters and I-V cures are presented in Figure 3a and Supporting Information (Figures S8–S11). Figure 3a shows the I-V curves and detailed parameters of the best-performing solar cell. The reverse scan curve shows the highest PCE (16.66%) and the forward scan curve indicates that the same cell achieved a PCE of 15.96%. The small difference between the two PCEs implies less hysteresis in the device prepared with 8 mg/mL MAI in NBA. In order to further study the performance of the cell under work conditions, the photocurrent density was measured at 0.833 V for 110 s. The stabilized photocurrent density was 18.38 mA/cm^2^, and a stable high PCE of 15.31% was obtained (Figure 3b). This stable power output efficiency suggests the possibility to prepare efficient and stable perovskite solar cells with 8 mg/mL MAI in NBA.

To check the *J*_sc_ of the best-performing cell, the external quantum efficiency (EQE) was measured (Figure 3c). The *J*_sc_ can be predicted by integrating the EQE spectrum with the incident photo flux density distribution using Equation (1),

Integrated *J*_SC_ = ʃ *qF*(*λF*(*λ*)EQ)d*λ* (1)

where *q* is the electron charge and *F*(*λ*) is the incident photon flux density (AM 1.5, ASTM G173) at wavelength λ. The integrated *J*_sc_ of the cell was 20.46 mA/cm^2^, which is close to the value obtained from the I–V measurement (Figure 3a). To investigate the reproducibility of the performance of cells fabricated using 8 mg/mL MAI in NBA, 40 individual devices were measured to obtain the corresponding PCEs. Statistical analysis of these PCEs showed an average value of 14.76% with a small standard deviation of 0.67% for all 40 cells (Figure 3d).

## 4. Conclusions

It was demonstrated that the solvent significantly affects the growth mechanism of perovskite grains. The morphology of perovskite films was controlled by manipulating the solvent polarity and concentration of MAI solutions. The use of solvents with long alkyl-chain molecule (i.e., low polarity solvent) allowed the MAPbI_3_ to grow via an *in situ* transformation step, which easily made the perovskite films compact, but increased the surface roughness due to the growth of unexpected nanorods/nanoplates. Using the solvent with a short alkyl chain (i.e., high polarity solvent) led to the dissolution-crystallization growth mechanism, resulting in rapidly generation of perovskite films with smooth surface, but with a number of pinholes. The best quality (highly compact, pinhole-free, and smooth) perovskite films were fabricated with an optimized MAI concentration of 8 mg/mL and NBA as the solvent, where both growth mechanisms worked together. The corresponding perovskite solar cell achieved a promising PCE of 16.66% and average PCE of 14.76% (for 40 cells). The present work may give some clues to other groups to further investigate the mechanisms of perovskite grain growth and prepare high-quality perovskite film through simple methods.

## Figures and Tables

**Table 1:** Morphologies of CH_3_NH_3_PbI_3_ (MAPbI_3_) films obtained from 6–12 mg/mL CH_3_NH_3_I (MAI) in four-selected solvents.

	Solvents	Isopropanol (IPA)	*n*-butyl Alcohol (NBA)	*n*-amyl Alcohol (NAA)	*n*-hexyl Alcohol (NHA)
Conentration					
6 (mg/mL)		Incomplete-covered	Incomplete-covered	compact with nanorods	compact with nanorods
8 (mg/mL)		Incomplete-covered	compact	compact with nanorods	compact with nanorods
10 (mg/mL)		Incomplete-covered	compact with nanorods	compact with nanorods and nanoplates	compact with nanorods and nanoplates
12 (mg/mL)		Incomplete-covered	compact with nanorods and nanoplates	compact with nanorods and nanoplates	compact with nanorods and nanoplates

## Supplementary Materials

Supplementary materials are available online at http://www.mdpi.com/2079-4991/8/11/897/s1.
