# Supplementary material for: A Compact and Smooth CH3NH3PbI3 Film: Investigation of Solvent Sorts and Concentrations of CH3NH3I towards Highly Efficient Perovskite Solar Cells
Source: Nanomaterials (Basel). 2018 Nov 1;8(11):897. doi: 10.3390/nano8110897 (PMC6265889; doi:10.3390/nano8110897)
Supplement: Supplementary file 1 [file nanomaterials-08-00897-s001.pdf]

Supporting Information

A Compact and Smooth $CH_3NH_3PbI_3$ Film: Investigation of Solvent Sorts and Concentrations of CH_3NH_3I towards Highly Efficient Perovskite Solar cells

Liang Chen*[a b], Hao Zhang[a], Jiyuan Zhang[c], Yong Zhou[b,c]

[a] *Hunan Key Laboratory of Applied Environmental Photocatalysis, Hunan Collaborative Innovation Center of Environmental and Energy Photocatalysis, Changsha University, 98 Hongshan Road, Changsha 410022, P.R. China.*

[b] *Jiangsu Key Laboratory for Nano Technology, National Laboratory of Solid State Microstructures, College of Engineering and Applied Sciences, and Collaborative Innovation Center of Advanced Microstructures, Nanjing University, 22 Hankou Road, Nanjing 210093, P.R. China.*

[c] *Kunshan Innovation Institute of Nanjing University, Kunshan, Jiangsu 215347, P. R. China.*

*Corresponding Author: Liang Chen ; *E-mail:* mg1122028@smail.nju.edu.cn;

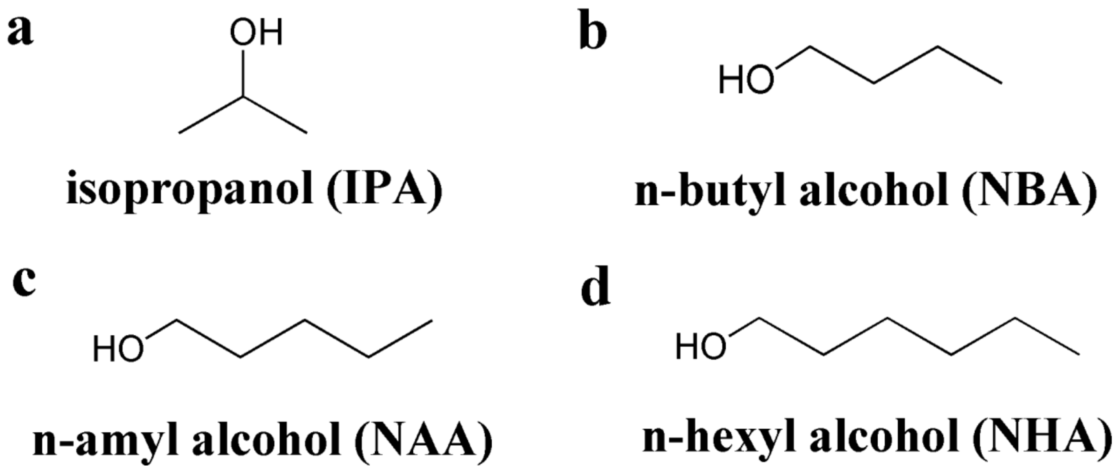

Figure S1. structural formula of the four-selected solvents molecule.

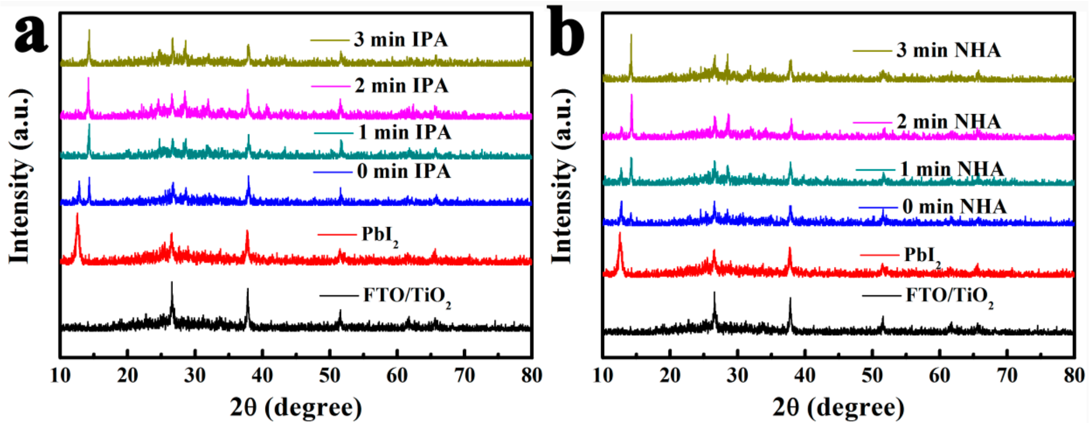

Figure S2. X-ray diffraction (XRD) patterns of Fluorine-doped tin oxide glass (FTO) substrate coated with TiO_2, PbI_2 film and the films prepared with loading time 0-3min using 6mg/mL MAI in (a) IPA and (b) NHA.

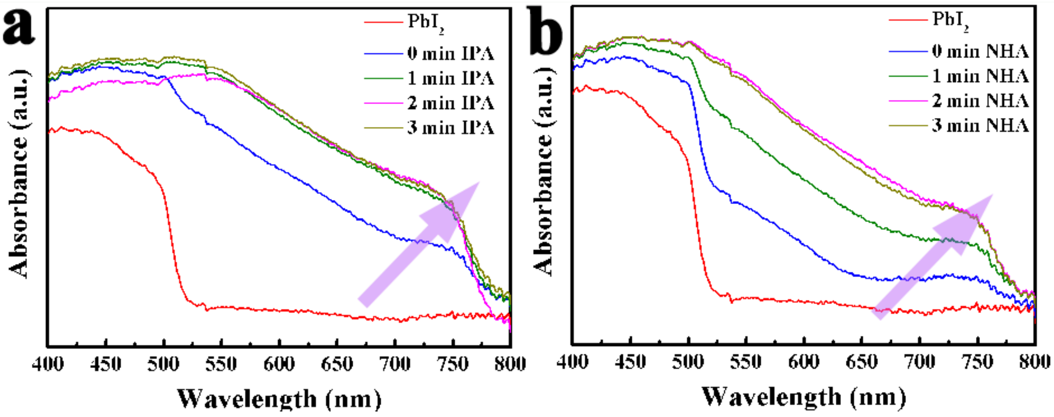

Figure S3. Ultraviolet–visible (UV-vis) absorption spectra of PbI_2 film and the films prepared with loading time 0-3min using 6mg/mL MAI in (a) IPA and (b) NHA.

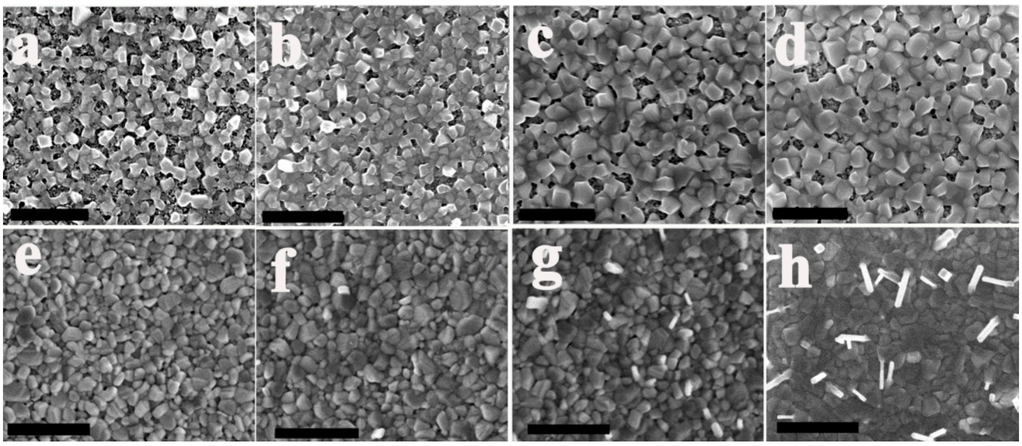

Figure S4. Field emission scanning electron microscope (FE-SEM) images of perovskite films prepared with loading time 0-3min using 6mg/mL MAI in (a)-(d) IPA and (e)-(h) NHA. The scale bars of a-h are 1μm.

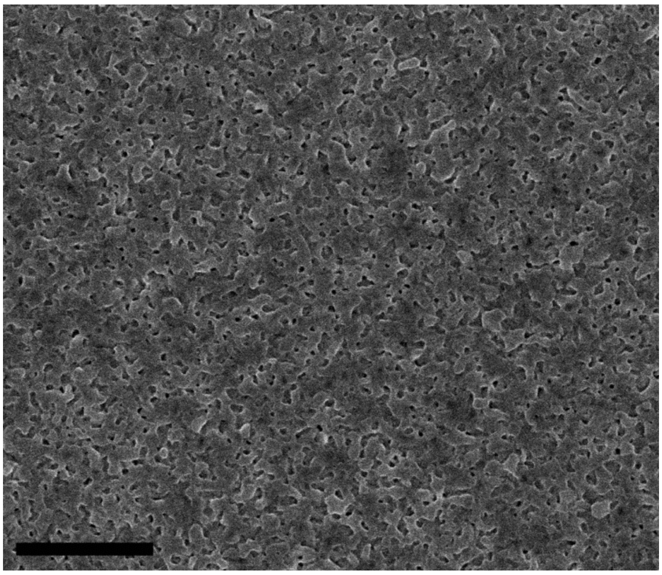

Figure S5. FE-SEM images of PbI_2 films. The scale bar is 1μm.

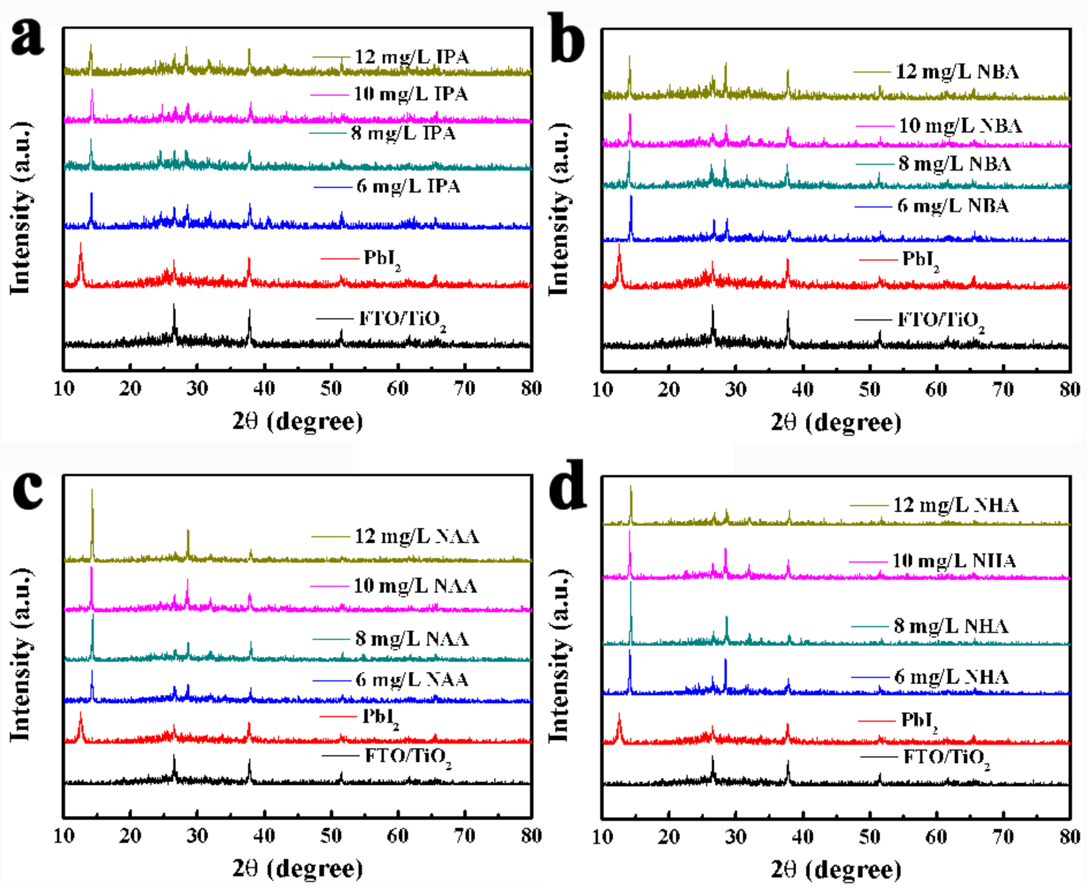

Figure S6. XRD patterns of FTO substrate coated withTiO_2, PbI_2 film and $MAPbI_3$ films obtained from 6-12 mg/mL MAI in (a) IPA, (b) NBA, (c) NAA and (d) NHA

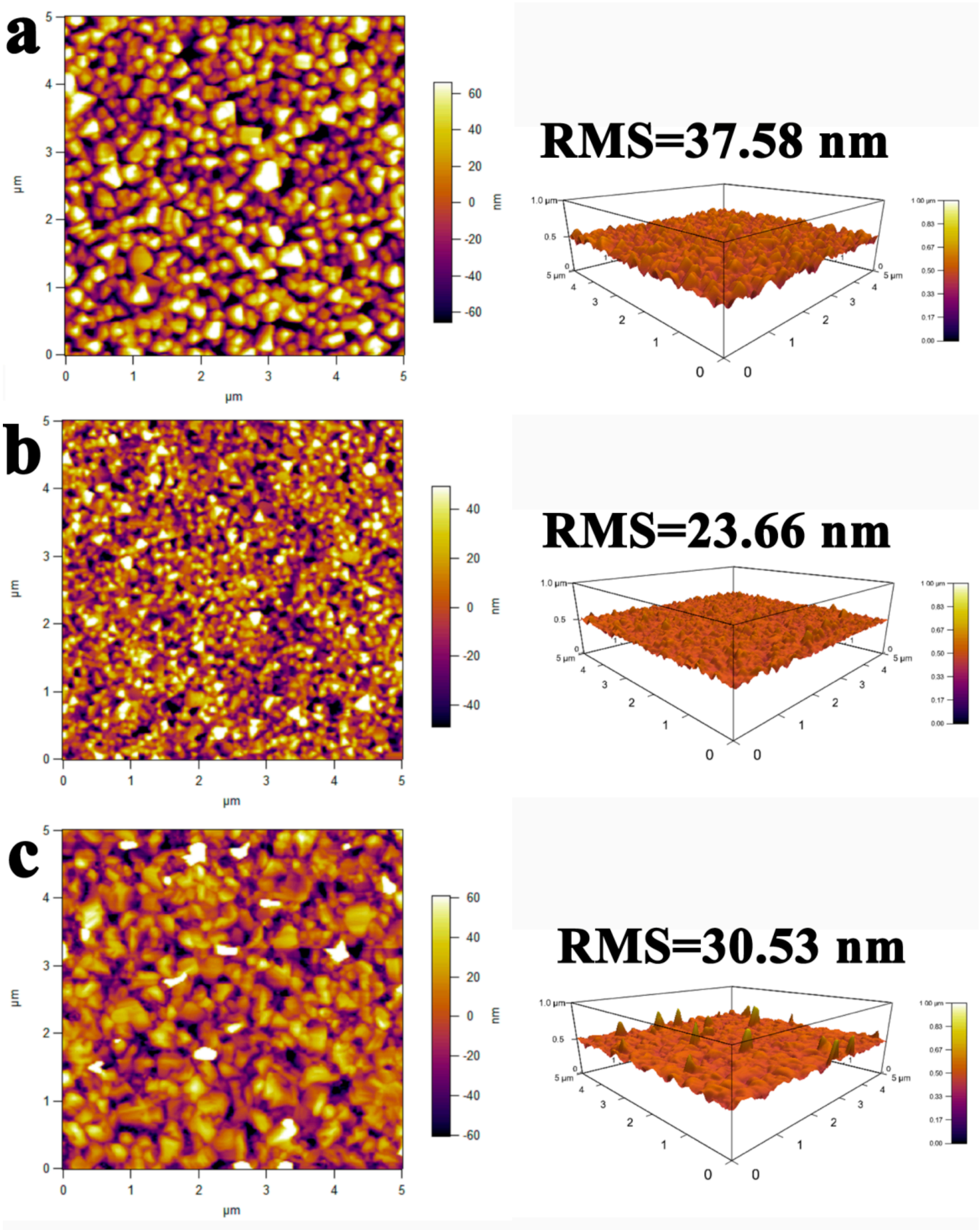

Figure S7. Atomic force microscope (AFM) images of three $MAPbI_3$ films basic types (a) incomplete-covered perovskite film, (b) compact perovskite film and (c) compact perovskite film with nanorods/nanoplates. a, b and c were obtained from 6, 8 and 10 mg/mL MAI in NBA, respectively.

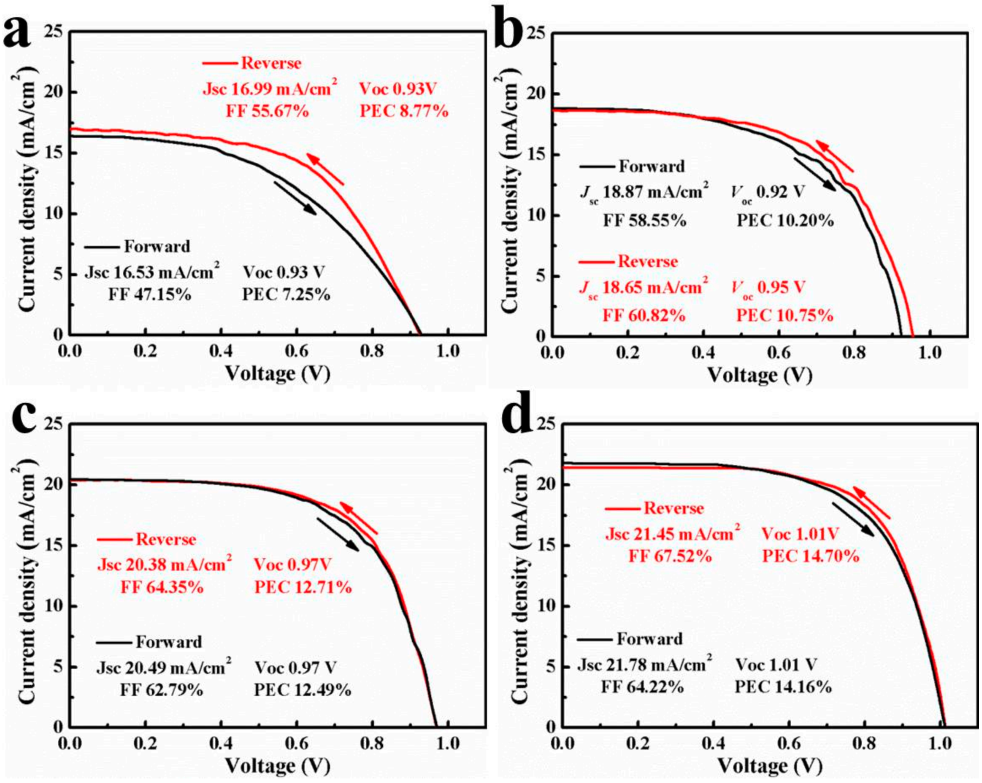

Figure S8. Detail parameters and I-V cures of the cells prepared by using (a) 6 mg/mL, (b) 8 mg/mL, (c) 10 mg/mL (d) 12 mg/mL MAI in IPA.

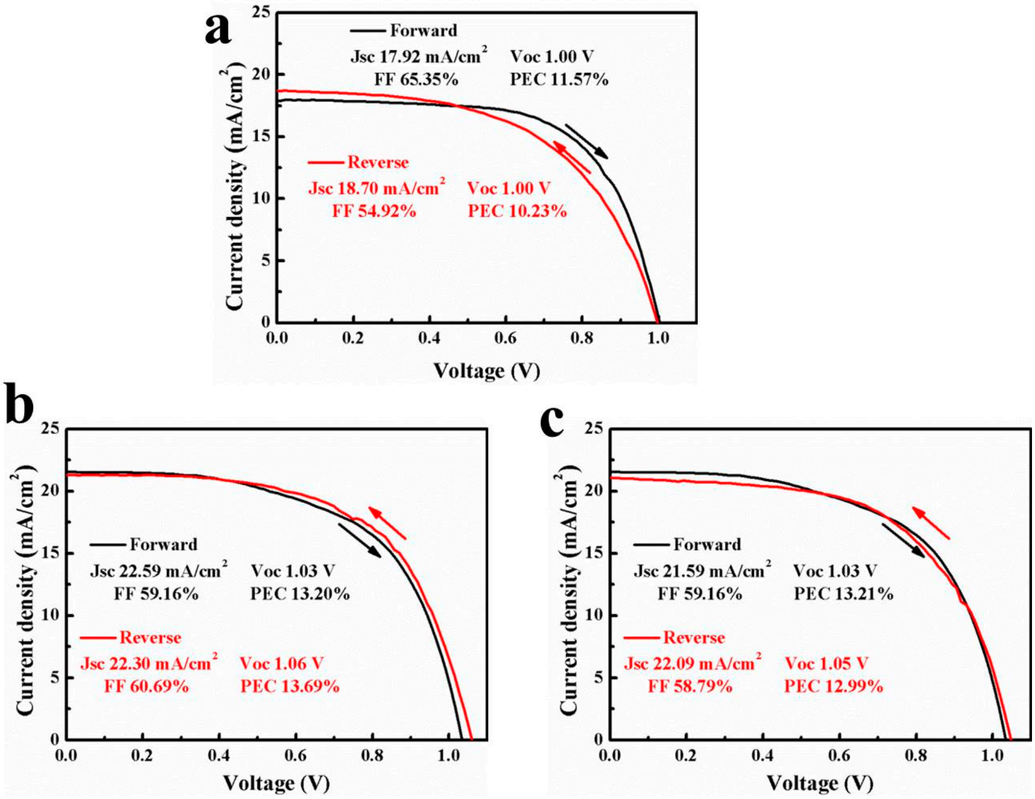

Figure S9. Detail parameters and I-V cures of the cells prepared by using (a) 6 mg/mL, (b) 10 mg/mL (c) 12 mg/mL MAI in NBA.

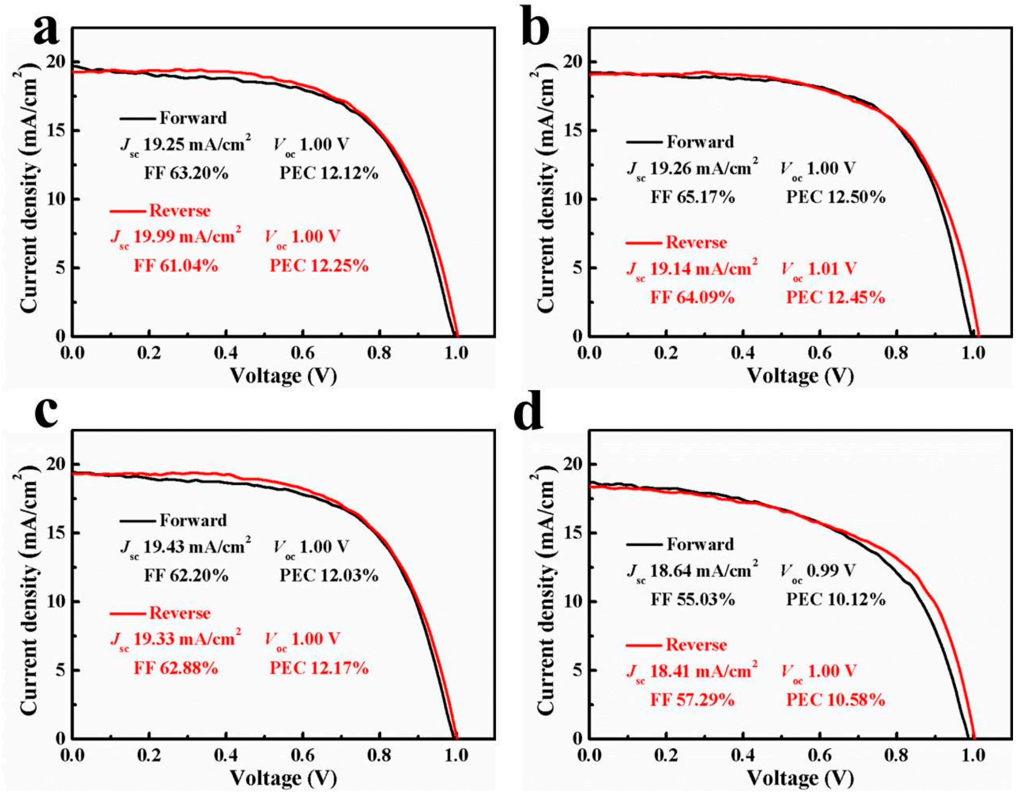

Figure S10. Detail parameters and I-V cures of the cells prepared by using (a) 6 mg/mL, (b) 8 mg/mL, (c) 10 mg/mL (d) 12 mg/mL MAI in NAA.

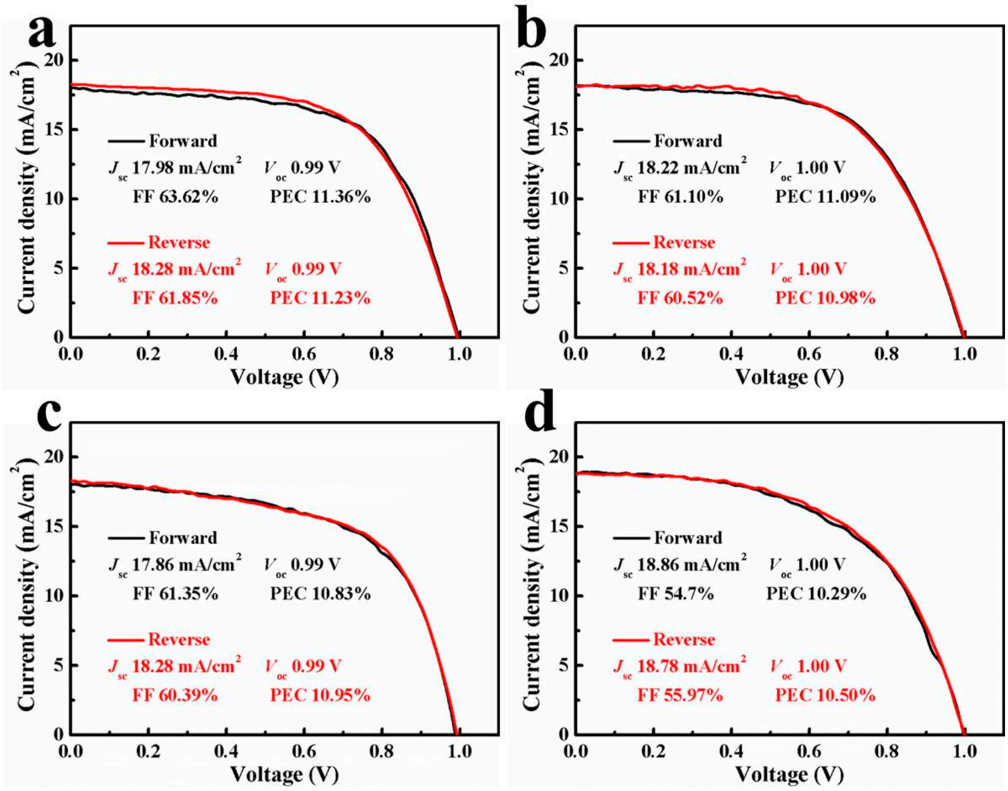

Figure S11. Detail parameters and I-V cures of the cells prepared by using (a) 6 mg/mL, (b) 8 mg/mL, (c) 10 mg/mL (d) 12 mg/mL MAI in NHA.

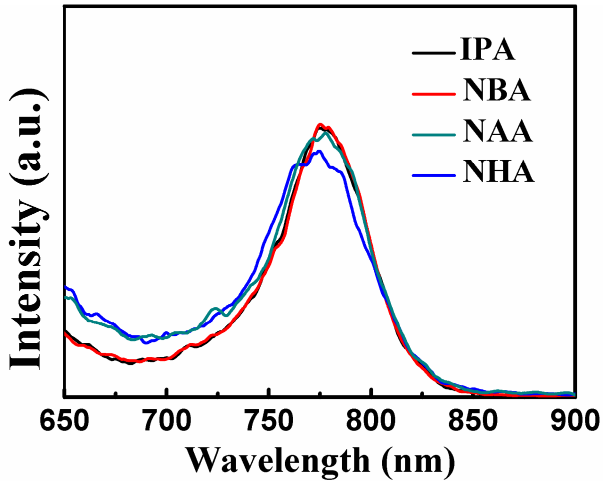

Figure S12. photoluminescence spectrum of $CH_3NH_3PbI_3$ perovskites films on FTO that were prepared by using 8 mg/mL MAI in IPA, NBA, NAA, and NHA.
